# Elasticity and Characteristic Stress Thresholds of Shale under Deep In Situ Geological Conditions

**DOI:** 10.3390/ma16196550

**Published:** 2023-10-04

**Authors:** Xiaofang Nie, Zidong Fan, Qin Zhou, Zilong Yao, Zheming Zhu, Li Ren

**Affiliations:** MOE Key Laboratory of Deep Earth Science and Engineering, College of Architecture and Environment, Sichuan University, Chengdu 610065, China; xfn@stu.scu.edu.cn (X.N.); fanzidong@stu.scu.edu.cn (Z.F.); zq937766477@outlook.com (Q.Z.); m17373147851_1@163.com (Z.Y.); zhemingzhu@hotmail.com (Z.Z.)

**Keywords:** shale, crack closure, crack initiation, elastic parameters, triaxial compression

## Abstract

The mechanical properties of shale are generally influenced by in situ geological conditions. However, the understanding of the effects of in situ geological conditions on the mechanical properties of shale is still immature. To address this problem, this paper provides insight into the elasticity and characteristic stress thresholds (i.e., the crack closure stress *σ*_cc_, crack initiation stress *σ*_ci_, and crack damage stress *σ*_cd_) of shales with differently oriented bedding planes under deep in situ geological conditions. To accurately determine the elastic parameters and crack closure and initiation thresholds, a new method—i.e., the bidirectional iterative approximation (BIA) method—which iteratively approaches the upper and lower limit stresses of the linear elastic stress-strain regime, was proposed. Several triaxial compression experiments were performed on Longmaxi shale samples under coupled in situ stress and temperature conditions reflecting depths of 2000 and 4000 m in the study area. The results showed that the peak deviatoric stress (*σ*_p_) of shale samples with the same bedding plane orientation increases as depth increases from 2000 m to 4000 m. In addition, the elastic modulus of the shale studied is more influenced by bedding plane orientation than by burial depth. However, the Poisson’s ratios of the studied shale samples are very similar, indicating that for the studied depth conditions, the Poisson’s ratio is not influenced by the geological conditions and bedding plane orientation. For the shale samples with the two typical bedding plane orientations tested (i.e., perpendicular and parallel to the axial loading direction) under 2000 and 4000 m geological conditions, the ratio of crack closure stress to peak deviatoric stress (*σ*_cc_/*σ*_p_) ranges from 24.83% to 25.16%, and the ratio of crack initiation stress to peak deviatoric stress (*σ*_ci_/*σ*_p_) ranges from 34.78% to 38.23%, indicating that the *σ*_cc_/*σ*_p_ and *σ*_ci_/*σ*_p_ ratios do not change much, and are less affected by the bedding plane orientation and depth conditions studied. Furthermore, as the in situ depth increases from 2000 m to 4000 m, the increase in *σ*_cd_ is significantly greater than that of *σ*_cc_ and *σ*_ci_, indicating that *σ*_cd_ is more sensitive to changes in depth, and that the increase in depth has an obvious inhibitory effect on crack extension. The expected experimental results will provide the background for further constitutive modeling and numerical analysis of the shale gas reservoirs.

## 1. Introduction

With the growing importance of mitigating environmental impacts and the increasing demand for clean energy sources, shale gas exploration has become a topic of great interest [1,2,3]. Since shale reservoirs have extremely low porosity and permeability [4], hydraulic fracturing treatment has been widely used to produce stimulated reservoir volume (SRV) [5]. From a rock mechanics perspective, both deformation and failure of shale formations occur during SRV treatments, and one of the challenges to the effective implementation and evaluation of SRV treatments is the currently incomplete understanding of the mechanical behavior of shale [6]. Detailed mechanical characterization of shales is essential for hydraulic fracturing stimulation design; specifically, to better predict and mitigate fracture closure after stimulation [7,8]. In addition, the high temperature and high confining pressure characteristics of in situ reservoirs make it difficult to understand the fracture properties of deep shales clearly [9,10], and the inherent anisotropy of shales also affects their mechanical properties [11,12,13]. Therefore, insight into the geomechanical properties of shales with differently oriented bedding planes under in situ stress conditions corresponding to different depths is important and highly welcomed.

Poisson’s ratio and the elastic modulus are the two dominant geomechanical parameters that determine the brittleness of shale gas formations, which defines the potential interval for hydraulic fracturing [14,15]. Knowledge of Poisson’s ratio and the elastic modulus also helps in defining the initiation conditions of hydraulic fractures [16]. Therefore, understanding the geomechanical parameters of shale reservoirs, especially Poisson’s ratio and the elastic modulus, which have a significant impact on fracture treatment performance, allows more accurate decisions to be made in the design and optimization of fracturing treatments for shale gas reservoirs. Many experimental studies have been reported on the elastic parameters of shale. For example, Hou et al. [17] and Masri et al. [18] revealed that as temperature increases, there is a significant decrease in the elastic modulus and compressive strength, accompanied by an increase in the overall deformability of the material. Abbas et al. [19] found that the strength parameters of shale increased significantly with higher confining pressures, while the elastic modulus varied slightly under different confining pressures. In contrast to the studies that examined only the individual effects of confining pressure or temperature on the mechanical properties of shale, Guo et al. [20] and Li et al. [21] focused on the coupling effect of both factors. The results of these studies show that increased temperature causes a gradual decrease in peak stress and modulus properties. It was also found that an increase in confining pressure results in a significant increase in peak stress, and that the elastic modulus also shows a tendency to increase. However, these studies did not relate the temperature and confining pressure to the actual deep in situ conditions to study the effects of different deep in situ geological conditions on shale elastic parameters.

During the shale gas extraction process, hydraulic fracturing reopens and/or creates fractures at various scales [22], and the rapid decline in production observed in such fractured reservoirs is mostly attributed to progressive fracture closure. According to Sheng et al. [23] and Zhao et al. [24], the three characteristic stress thresholds (i.e., the crack closure, crack initiation, and crack damage stress thresholds) represent the different stages of deformation and failure. Therefore, it is of great importance to determine the characteristic stresses to study the deformation and failure process of shale. Different experimental techniques have been applied in evaluating the characteristic stresses of shale gas reservoirs, including uniaxial and triaxial compressions. For example, He et al. [6] performed several uniaxial compression experiments on Longmaxi shale samples with different bedding plane orientations, and the results showed that with an increase in the bedding plane angle, the crack initiation stress decreases and then increases. Concerning the in situ stress state, Li et al. [25] performed triaxial tests on shale samples with seven different bedding plane orientations (0°, 15°, 30°, 45°, 60°, 75° and 90°). They concluded that the crack initiation stress is independent of the bedding plane orientation, while the anisotropic characteristics of the shale strongly affect the crack damage stress. Furthermore, Lu, et al. [26] considered the coupling effect of temperature and confining pressure on the mechanical properties of shales. They conducted triaxial compression tests on Longmaxi shales with two typical bedding plane orientations (0° and 90°) under a high temperature (90.93 °C) and confining pressure (69.82 MPa) corresponding to a depth of 3000 m. That study showed that the crack initiation stress and crack damage stress were higher for a bedding plane orientation of 90° than for that of 0°. However, few existing studies have investigated the mechanical properties of shale under in situ stress conditions coupled with high-temperature and high-pressure conditions. Furthermore, few existing studies have investigated the effect of in situ geological conditions at different depths on the mechanical properties of shale.

To investigate the effect of in situ conditions at different depths on the elastic properties of shale, in this research, several triaxial compression tests were performed at high temperatures and confining pressures to simulate different depths. In addition, a novel method for determining crack closure and initiation thresholds was proposed, which can accurately determine the elastic modulus and Poisson’s ratio. Therefore, the strength characteristics, fracture patterns, elastic parameters, and characteristic stresses (i.e., *σ*_cc_, *σ*_ci_, and *σ*_cd_) of Longmaxi shale with two typical bedding plane orientations (i.e., perpendicular and parallel to the axial loading direction) under different depth conditions were analyzed. In addition, a series of characteristic stress to peak deviatoric stress ratios (i.e., *σ*_cc_/*σ*_p_, *σ*_ci_/*σ*_p_, and *σ*_cd_/*σ*_p_) were investigated for each shale sample. The results can help to determine the geomechanical parameters of gas shale reservoirs for the development of hydraulic fracturing and will provide the background for further constitutive modeling and numerical analysis of the shale gas reservoirs.

## 2. Methods

### 2.1. Theoretical Methods

**Figure 1:**
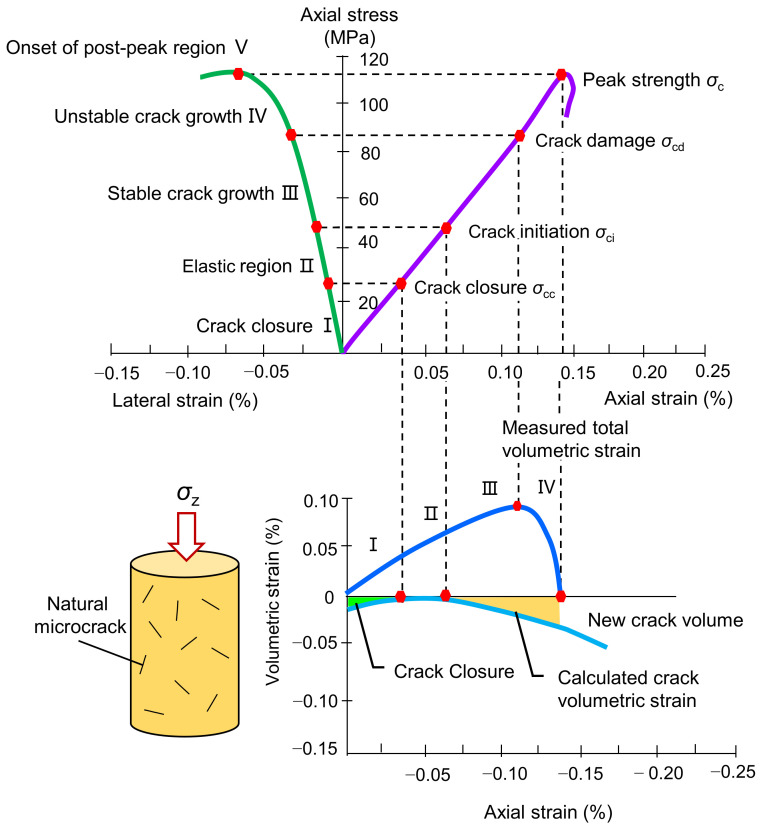
Stress-strain diagram showing the stages of microcrack development (after [6,29]).

To study the progressive damage evolution behavior of rocks, the experimental results indicate that the rock failure process can be divided into five stages: the closure stage, elastic deformation stage, crack stable growth stage, crack unstable growth stage, and postpeak stage [27,28,29,30]. The measurable stresses associated with these stages—namely, the crack closure stress (*σ*_cc_), crack initiation stress (*σ*_ci_), crack damage stress (*σ*_cd_), and peak stress (*σ*_c_)—are shown in Figure 1. When the applied stress exceeds the crack initiation stress, the linear elastic relation of the tested rock ends. Therefore, accurate identification of the crack closure and initiation thresholds is the key to accurately obtaining the linear elastic portion of the stress-strain curve, and is thus also key to determining the elastic parameters. Methods for determining crack closure and initiation thresholds under compression are primarily dependent on the measured strains. These methods are reviewed below.

#### 2.1.1. Brief Review of Crack Closure and Initiation Determination Methods

(a) Crack volumetric strain (CVS) method

Martin et al. [28] conducted an extensive experimental study on the Lac du Bonnet granite. With the strength properties of the rock, they proposed that *σ*_ci_ could be determined by plotting stress–crack volumetric strain curves. *σ*_ci_ corresponds to the stress at the end of the horizontal section of the stress–crack volumetric strain plot where the volumetric strain of the crack is equal to zero, as shown in Figure 1. Compared to methods that interpret cracking points using manual tangent lines, the CVS method is more accurate and objective. However, the CVS method can easily lead to errors in the determination of the point that deviates from the horizontal section. In addition, elastic volumetric strain is used in the CVS method for the determination of crack initiation stress, which is also determined by the elastic modulus and Poisson’s ratio. Therefore, to accurately determine the crack initiation stress using the CVS method, it is crucial to know the accurate values of the elastic modulus and Poisson’s ratio in advance. These elastic parameters are typically determined as the secant values from the range of 0.01% of the lateral strain to half of the peak strength [31], and the secant values from a linear stage of the stress-strain curve [32], respectively. However, these methods are generally empirical, and the results obtained should be validated. In addition, the nonlinearity of the lateral strain response complicates the measurement of Poisson’s ratio. Therefore, the elastic parameters have a great influence on the *σ*_ci_ determined by the CVS method. In particular, the crack volumetric strain is sensitive to Poisson’s ratio [29].

(b) Iterative method

**Figure 2:**
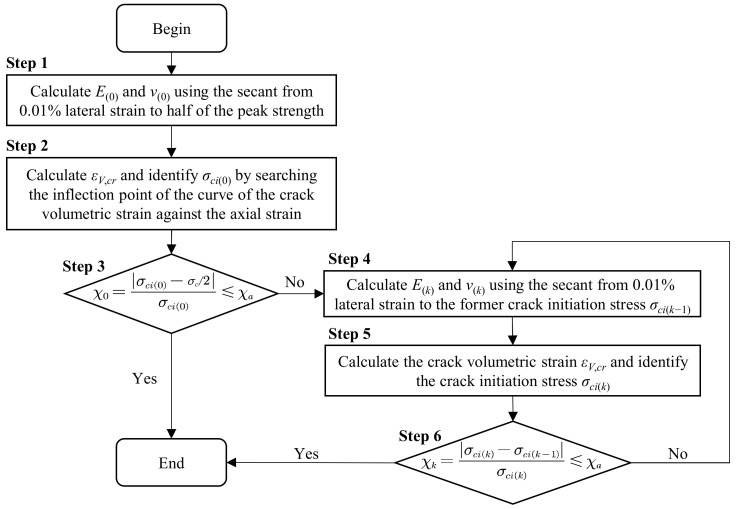
Flow chart of crack initiation stress and elastic parameter determination [6].

To overcome the limitations of the empirical crack initiation stress threshold, He et al. [6] proposed an iterative method that synchronously determines the elastic parameters and the crack initiation stress, based on the work of [31]. The iterative method determines the elastic parameters and crack initiation stress step by step over a stress range from 0.01% of the lateral strain to a variable upper limit stress, and the detailed procedure is shown in Figure 2. This method assumes that the stress-strain curve of the rock sample enters the linear stage before 0.01% of the lateral strain, and always takes the stress corresponding to 0.01% of the lateral strain as the crack closure stress for the iterative calculation. However, for rocks with an obvious compaction stage, the stress-strain curve still has a concave shape at 0.01% of the lateral strain, which thus cannot be the starting point of the linear section. In addition, always assuming the stress corresponding to 0.01% of the lateral strain is the crack closure stress in the iterative method causes the Poisson’s ratio to be quite different from the real Poisson’s ratio. In addition, the iterative method still adopts the selection of the inflection point of the axial strain-crack volumetric strain curve as the crack initiation stress, which is highly influenced by subjectivity.

(c) Volumetric stiffness method (VSM)

**Figure 3:**
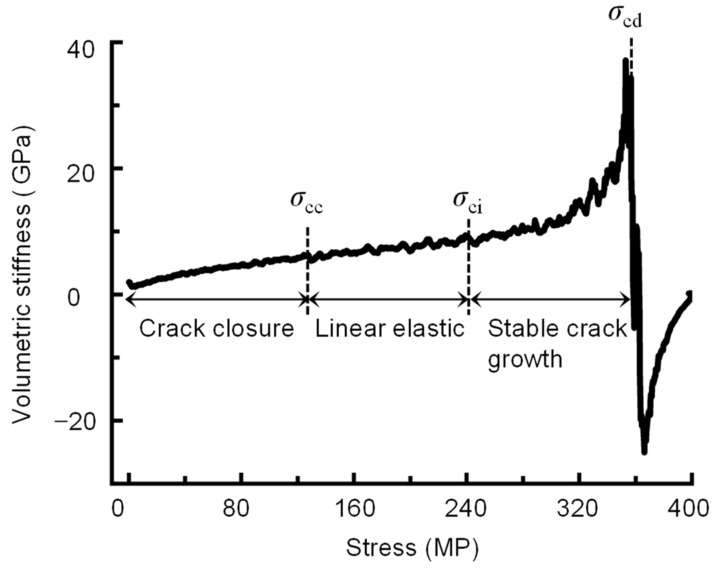
VSM for determining characteristic stresses (after [29]).

Eberhardt et al. [29] proposed a VSM to determine crack initiation and damage stresses based on the shape of the stress-volumetric strain curve (slope of the stress-volumetric strain curve) obtained by the moving point regression technique at certain intervals of the data. Their results indicate that the size of the regression interval should be approximately 3% of the total number of x and y data pairs [29]. After the initial irregular region, the stress-volumetric strain curve entered into a linear region, at which point the stress was the crack closure stress *σ*_cc_. Then, the curve transitioned from a linear to an irregular region without any discontinuous change in slope, and the stress at this point was defined as the crack initiation stress *σ*_ci_. The stress when the stress-volumetric strain curve begins to sharply drop was defined as the crack damage stress *σ*_cd_. The detailed principle is shown in Figure 3. The VSM can obtain the rock characteristic stresses without calculating Poisson’s ratio and the elastic modulus; thus, it can be applied in situations where Poisson’s ratio cannot be obtained. However, the VSM relies on visual observation to select the inflection point, resulting in a high degree of error and subjectivity.

#### 2.1.2. Bidirectional Iterative Approximation (BIA) Method

During the propagation of the microcracks inside a rock sample, the total volumetric strain *ε*_V_ can be calculated as [6]:

(1) $$\varepsilon_{V}=\varepsilon_{V,e}+\varepsilon_{V,\mathrm{cr}}$$

where *ε*_V,e_ is the elastic strain of the rock matrix, and *ε*_V,cr_ is the crack volumetric strain induced by crack deformation. For an isotropic rock sample under triaxial compression, the total volumetric strain *ε*_V_ and elastic volumetric strain *ε*_V,e_ can be written as:

(2) $$\varepsilon_{V}=\varepsilon_{1}+2\varepsilon_{3},$$

(3) $$\varepsilon_{V,e}=\frac{1-2\upsilon }{E}(\sigma_{1}-\sigma_{3}),$$

where *ε*_1_ is the axial strain, *ε*_3_ is the lateral strain, *E* is the elastic modulus, *ν* is Poisson’s ratio, *σ*_l_ is the axial stress, and *σ*_3_ is the confining pressure. Noting Equations (1)–(3), the crack volumetric strain *ε*_V,cr_ can then be calculated by [33]

(4) $$\varepsilon_{V,\mathrm{cr}}=\varepsilon_{1}+2\varepsilon_{3}-\frac{1-2\upsilon }{E}(\sigma_{1}-\sigma_{3}).$$

**Figure 4:**
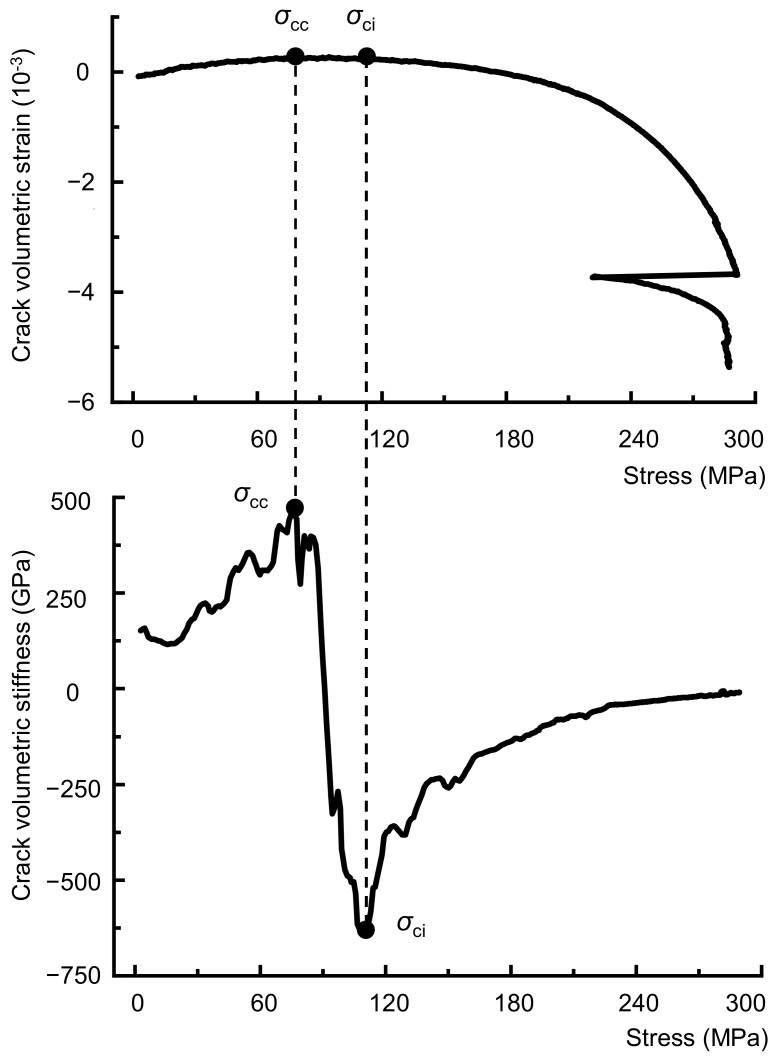
Determining the crack closure and initiation thresholds using the stress-crack volumetric stiffness curve.

**Figure 5:**
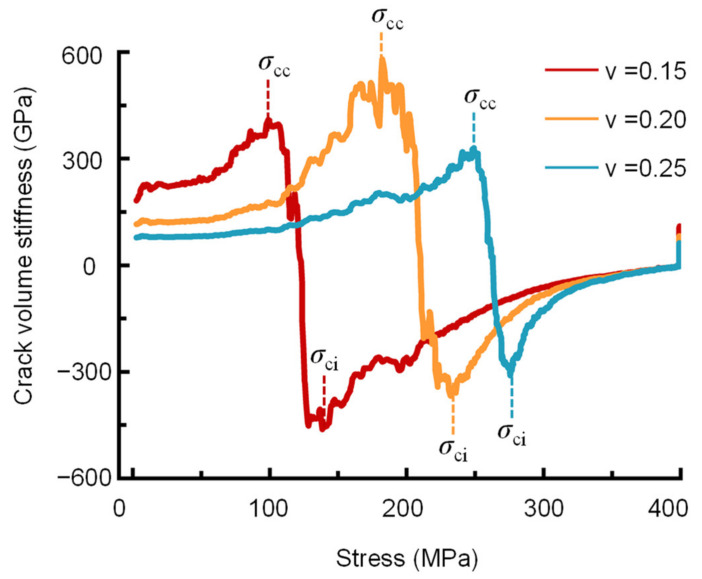
Variability in crack volume stiffness reversal with Poisson’s ratio.

According to the above equations, the crack volumetric strain of a rock can be obtained. Based on the moving point regression technique, the stress-crack volume stiffness curve (slope of the stress- crack volumetric strain curve) can also be used to determine the crack closure and initiation thresholds [29]. The rock fracture network can be viewed as many individual crack bodies. As the load increases, the crack bodies are continuously forced to close, resulting in a gradual increase in the stiffness of the crack bodies. When the crack bodies completely close, their stiffness increases to a peak, and the stress is the crack closure stress. After the linear elastic region, rock samples do not behave elastically, and stress adjustment occurs inside the crack body. Therefore, when the stress-crack volume stiffness curve approaches its minimum crack volume stiffness (which is negative), this indicates the initiation of cracks. The stress at this point is known as the crack initiation stress, *σ*_ci_. As the load increases, the stiffness of the crack body gradually decreases until the rock is damaged. When the rock fails, the stiffness of the crack body approaches zero (Figure 4). However, the crack initiation stress is very sensitive to Poisson’s ratio, and a change of ±0.05 in Poisson’s ratio can cause a ± 40% change in the *σ*_cc_ and *σ*_ci_ values, as shown in Figure 5 and Table 1.

**Figure 6:**
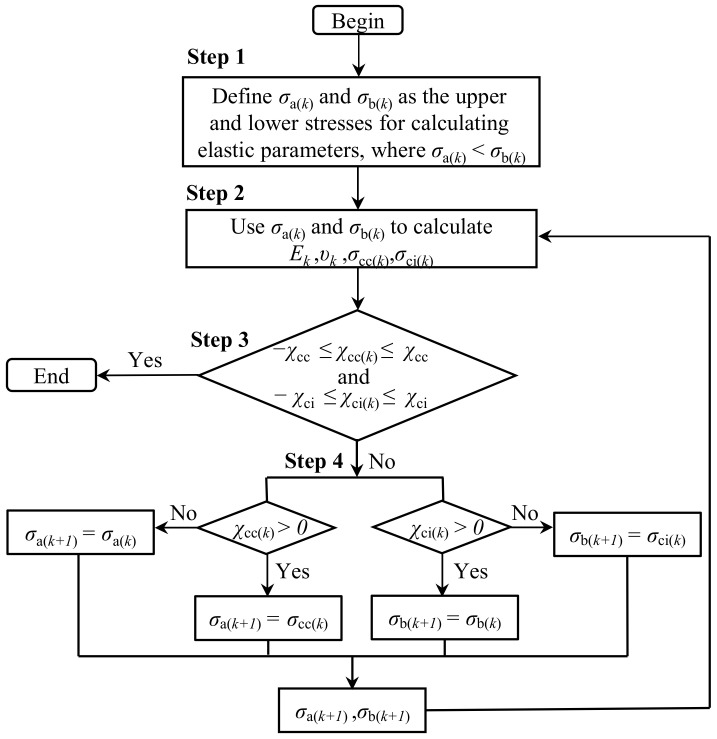
Flow chart of crack closure and crack initiation thresholds and elastic parameter determination using the BIA method.

**Figure 7:**
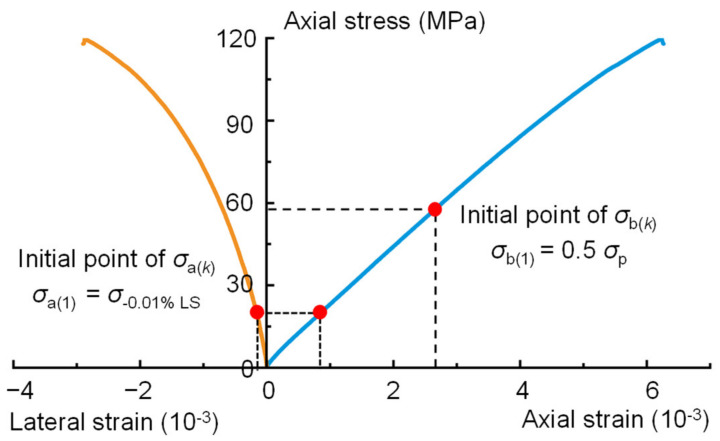
Determination of the initial values of the lower limit stress *σ*_a(*k*)_ and upper limit stress *σ*_b(*k*)_.

To avoid the influence of the subjectivity and accuracy of the elastic parameter values on the crack initiation stress, a new method—the bidirectional iterative approximation (BIA) method—was presented. This BIA method is based on the work of [6,29], which can simultaneously determine the elastic parameters, the crack closure threshold, and the initiation threshold. The detailed procedure (as shown in Figure 6) is described as follows:

Step 1: Determine the initial values of the lower limit stress *σ*_a(*k*)_ and upper limit stress *σ*_b(*k*)_. *σ*_a(*k*)_ is the lower limit stress, and *σ*_b(*k*)_ is the upper limit stress, which defines the stress range used to calculate the elastic parameters of the rock. Consider 0.01% of the lateral strain to half the peak strength (0.5*σ*_p_) as the linear elastic region to calculate the initial elastic modulus *E*_(0)_ and Poisson’s ratio *ν*_0_. This means that when k = 1, *σ*_a(1)_ is equal to the stress corresponding to 0.01% of the lateral strain, and *σ*_b(1)_ = 0.5*σ*_p_, as shown in Figure 7.

Step 2: Calculate *σ*_cc(*k*)_ and *σ*_ci(*k*)_. The elastic modulus *E*_k_ is determined as the secant value in the range of the lower limit stress *σ*_a(*k*)_ to the upper limit stress *σ*_b(*k*)_. In addition, Poisson’s ratio *ν*_k_ is equal to the negative lateral strain difference divided by the axial strain difference between *σ*_a(*k*)_ and *σ*_b(*k*)_. Substitute *E*_k_ and *ν*_k_ into Equation (4) to calculate the crack volumetric strain *ε*_V,cr_, and then plot the stress-crack volumetric stiffness curve. Determine the stress corresponding to the highest points of the stress-crack volumetric stiffness curve as the crack closure stress *σ*_cc(*k*)_, and the lowest point of the curve as the crack initiation stress *σ*_ci(*k*)_, as depicted in Figure 4.

Step 3: Observe whether the current errors (*χ*_cc(*k*)_ and *χ*_ci(*k*)_) are less than or equal to the tolerable errors (*χ*_cc_ and *χ*_ci_). The current error *χ*_cc(*k*)_ between *σ*_cc(*k*)_ and *σ*_a(*k*)_ is calculated by Equation (5), and the current error *χ*_ci(*k*)_ between *σ*_ci(*k*)_ and *σ*_b(*k*)_ is calculated by Equation (6); i.e.,

(5) $$\chi_{\mathrm{cc}(k)}=\frac{\sigma_{\mathrm{cc}(k)}-\sigma_{a(k)}}{\sigma_{a(k)}},$$

(6) $$\chi_{\mathrm{ci}(k)}=\frac{\sigma_{\mathrm{ci}(k)}-\sigma_{b(k)}}{\sigma_{b(k)}}.$$

The tolerable errors (*χ*_cc_ and *χ*_ci_) can be set to 1% or 2% according to the accuracy requirements. Observe whether the current errors (*χ*_cc(*k*)_ and *χ*_ci(*k*)_) are less than or equal to the tolerable errors (*χ*_cc_ and *χ*_ci_) using Equations (7) and (8):

(7) $$-\chi_{\mathrm{cc}}\leq \chi_{\mathrm{cc}(k)}\leq \chi_{\mathrm{cc}},$$

(8) $$-\chi_{\mathrm{ci}}\leq \chi_{\mathrm{ci}(k)}\leq \chi_{\mathrm{ci}}$$

If they are, end the calculations, and consider that the period between *σ*_a(*k*)_ and *σ*_b(*k*)_ is the elastic deformation phase of the rock, so the determined elastic parameter (*E*_k_ and *ν*_k_), crack closure stress (*σ*_cc(*k*)_), and crack initiation stress (*σ*_ci(*k*)_) results are reliable. Otherwise, the stage from the crack closure stress (*σ*_cc(*k*)_) to the crack initiation stress (*σ*_ci(*k*)_) calculated in this step is not the elastic stage of the stress-strain curve, so proceed to step 4. This means that if Equations (7) and (8) are not both correct, the iterative calculations in both directions should be continued so that the stages of *σ*_cc(*k*)_ and *σ*_ci(*k*)_ continue to approach the elastic stage of the stress-strain curve.

**Figure 8:**
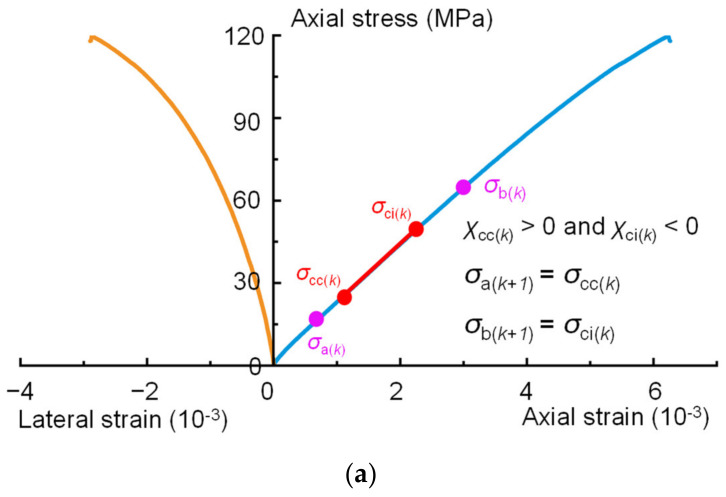

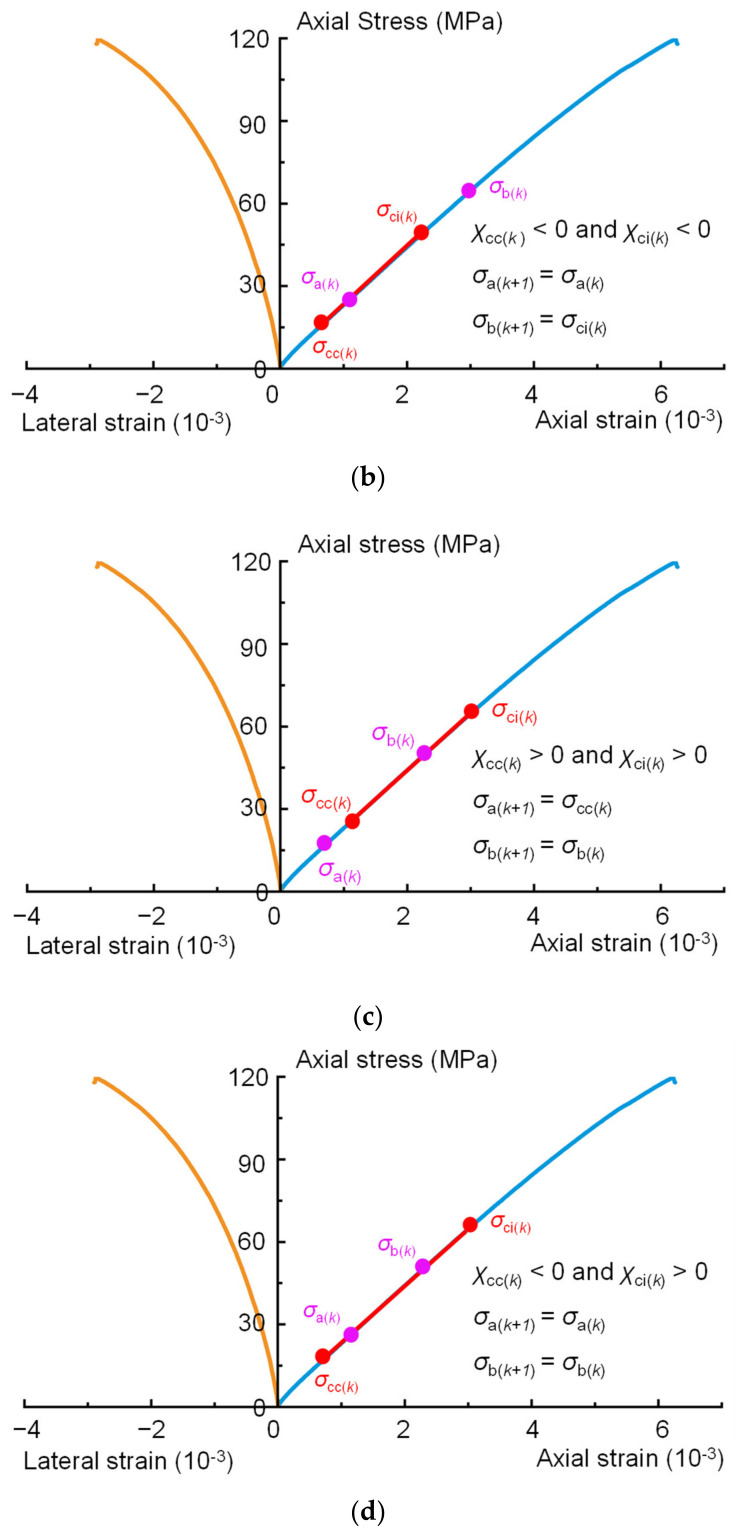
Schematic of the determination of *σ*_a(*k+1*)_ and *σ*_b(*k+1*)_: (**a**) when *χ*_cc(*k*)_ > 0 and *χ*_ci(*k*)_ < 0, *σ*_a(*k+1*)_ = *σ*_cc(*k*)_ and *σ*_b(*k+1*)_ = *σ*_ci(*k*)_; (**b**) when *χ*_cc(*k*)_ < 0 and *χ*_ci(*k*)_ < 0, *σ*_a(*k+1*)_ = *σ*_a(*k*)_ and *σ*_b(*k+1*)_ = *σ*_ci(*k*)_; (**c**) when *χ*_cc(*k*)_ > 0 and *χ*_ci(*k*)_ > 0, *σ*_a(*k+1*)_ = *σ*_cc(*k*)_ and *σ*_b(*k+1*)_ = *σ*_b(*k*)_; (**d**) when *χ*_cc(*k*)_ < 0 and *χ*_ci(*k*)_ > 0, *σ*_a(*k+1*)_ = *σ*_a(*k*)_ and *σ*_b(*k+1*)_ = *σ*_b(*k*)_.

Step 4: Determine *σ*_a(*k+1*)_ and *σ*_b(*k+1*)_ to be used for the (k + 1) iterations of the calculation. For the (k + 1) iterative calculations, *σ*_a(*k+1*)_ and *σ*_b(*k+1*)_ are selected from *σ*_a(*k*)_, *σ*_b(*k*)_, *σ*_cc(*k*)_, and *σ*_ci(*k*)_, which are used to approach the linear portion of the stress-strain curve. If the current error *χ*_cc(*k*)_ calculated by Equation (5) is greater than 0, it means that *σ*_cc(*k*)_ is greater than *σ*_a(*k*)_, and *σ*_cc(*k*)_ is closer to the linear portion of the stress-strain curve. Therefore, *σ*_cc(*k*)_ should be selected for the next calculation (Figure 8a or Figure 8c); i.e., *σ*_a(*k+1*)_ = *σ*_cc(*k*)_. Otherwise, *σ*_a(*k*)_ should be selected for the next calculation (Figure 8b or Figure 8d); i.e., *σ*_a(*k+1*)_ = *σ*_a(*k*)_. Follow the same method to determine the value of *σ*_b(*k+1*)_. If the error *χ*_ci(*k*)_ calculated by Equation (6) is greater than 0, *σ*_ci(*k*)_ is greater than *σ*_b(*k*)_, and *σ*_b(*k*)_ is closer to the linear portion of the stress-strain curve. Therefore, *σ*_b(*k*)_ should be selected for the next calculation (Figure 8c or Figure 8d); i.e., *σ*_b(*k+1*)_ = *σ*_b(*k*)_. Otherwise, *σ*_ci(*k*)_ should be selected for the next calculation (Figure 8a or Figure 8b); i.e., *σ*_b(*k+1*)_ = *σ*_ci(*k*)_.

It should be noted that the use of one approach to determine the *σ*_ci_ of brittle rocks may not be sufficient. The BIA method is based on the stress-strain curve, which depends on the measurement of axial and lateral strains. Due to the heterogeneity of the rock, localized strain development can lead to biased strain measurement results. Therefore, several methods should be used to increase the reliability of the *σ*_ci_ values obtained, such as the AE technique.

### 2.2. Experimental Method

**Figure 9:**
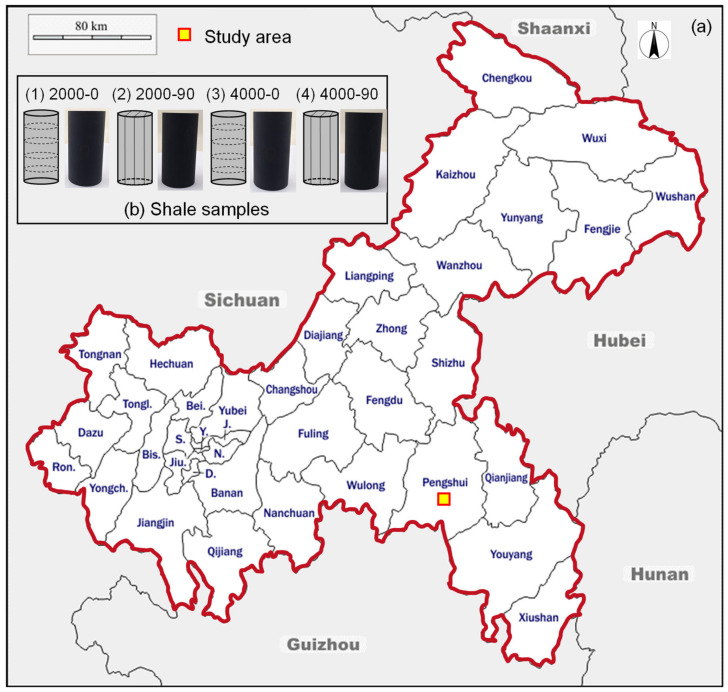
(**a**) Location of the study area in South China; (**b**) Shale samples that were taken from the study area. For 2000-0, 2000 is the depth of 2000 m, and 0 is the bedding plane angle *β* = 0°.

The shale material was selected from an outcrop of the Silurian Longmaxi Formation in the Chongqing region of Southwest China. The location of the study area is shown in Figure 9a, and the shale samples taken from the study area are shown in Figure 9b. In this study, shale samples were collected with two typical bedding plane orientations, perpendicular to the axial loading direction (*β* = 0°) and parallel to the axial loading direction (*β* = 90°). Two samples were taken for each bedding plane orientation, for a total of four samples. According to the International Society for Rock Mechanics (ISRM) standard for rock sample preparation [34,35], cylindrical samples with a height of 100 mm and a diameter of 50 mm were prepared as shown in Figure 9b. To investigate the effect of different depths of in situ conditions on the mechanical properties of shale, triaxial compression tests were performed using the MTS 815 rock mechanics testing system. Shale reservoirs in China are mainly concentrated at depths of 2500~4500 m [8]. The high-temperature and high-confining pressure conditions are determined based on the in situ conditions at depths of 2000 m and 4000 m. The corresponding temperature at a depth of 2000 m is 73.49 °C, while the confining pressure is 42.64 MPa. On the other hand, at a depth of 4000 m, the corresponding temperature is 102.7 °C, while the confining pressure is 89.15 MPa, as shown in Table 2. During the test, the temperature was controlled at a heating rate of 12 °C/h. After reaching and stabilizing the target temperature, the confining pressure corresponding to the depth of the occurrence environment was applied at a rate of 3 MPa/min. Finally, the displacement control method was used to load the shale sample to failure at a loading rate of 0.04 mm/min.

## 3. Insight into the Mechanical Properties of Shale under Deep In Situ Conditions

### 3.1. Strength Characteristics

**Figure 10:**
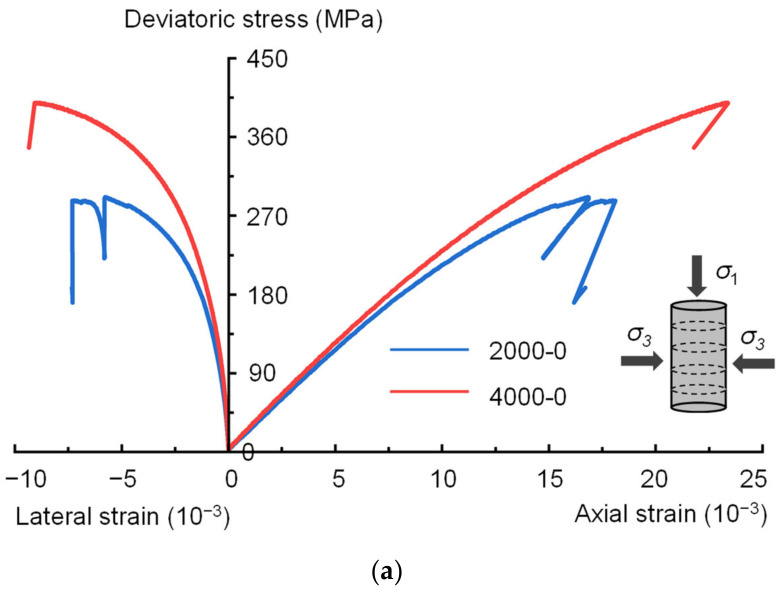

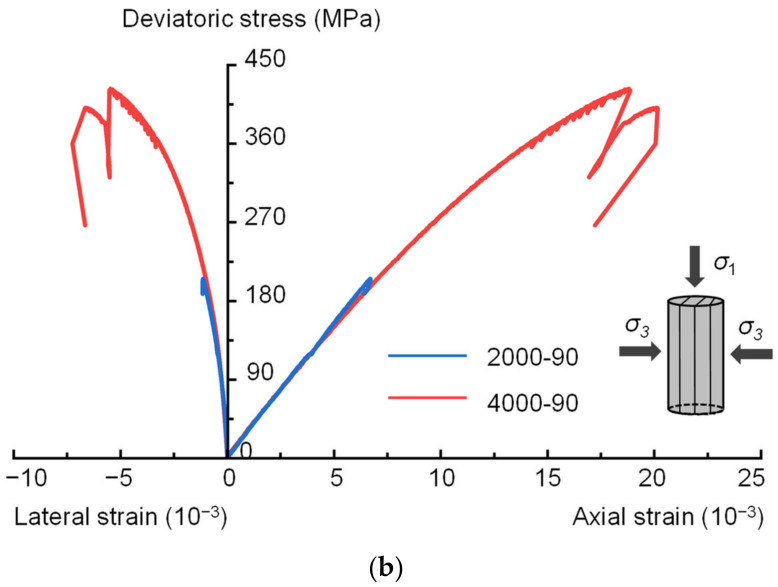
Stress-strain curve of shale samples: (**a**) bedding plane angle *β* = 0°; (**b**) bedding plane angle *β* = 90°.

Stress-strain curves of shale samples subjected to triaxial compression tests show obvious brittle characteristics (Figure 10). The peak deviatoric stresses of the 2000-0, 4000-0, 2000-90 and 4000-90 samples are 291.35, 399.47, 205.51, and 422.97 MPa, respectively. Shale samples with different bedding plane orientations have different strengths under the same deep in situ geological conditions. At a depth of 2000 m, the peak deviatoric stress of the shale sample with *β* = 0° is 41.77% higher than that of the sample with *β* = 90°. However, as the depth increased to 4000 m, the peak deviatoric stress of the *β* = 0° sample differed by less than 6% from that of the *β* = 90° sample. This difference may be because at a depth of 2000 m, the cracks in the sample with the loading direction parallel to the bedding plane orientation (*β* = 90°) tend to propagate along the vertical weak bedding surface, resulting in the strength of the sample with *β* = 90° being significantly lower than that of the sample with *β* = 0°. As the depth increases to 4000 m, the confining pressure increases, and the inhibitory effect on the cracks of samples with different bedding plane orientations remains consistent, so that the peak deviatoric stresses of the sample with *β* = 90° and the sample with *β* = 0° are similar. On the other hand, for shale samples with the same bedding plane orientations, the peak deviatoric stresses increase significantly with increasing depth. However, the degree of increase in strength with increasing depth is different for shale samples with different bedding plane orientations. As the depth increases from 2000 m to 4000 m, the peak deviatoric stresses of shale samples with *β* = 0° and *β* = 90° increase by 37.11% and 105.81%, respectively. This is because the cracks extending along the vertical weak laminar surfaces in the *β* = 90° samples are more sensitive to the change in confining pressure, leading to a larger difference in the strength of the samples in the different depth cases.

### 3.2. Elastic Parameters

**Figure 11:**
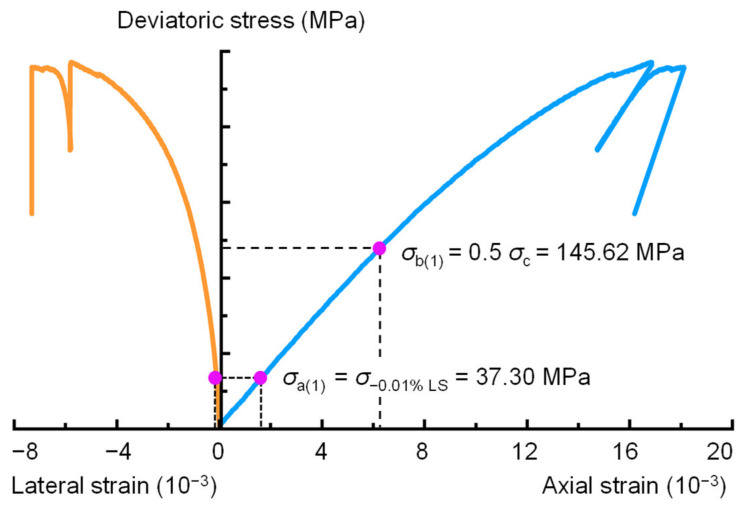
Definition of *σ*_a(1)_ and *σ*_b(1)_ for the calculation of the crack closure and initiation stresses of the 2000-0 sample using the BIA method.

**Figure 12:**
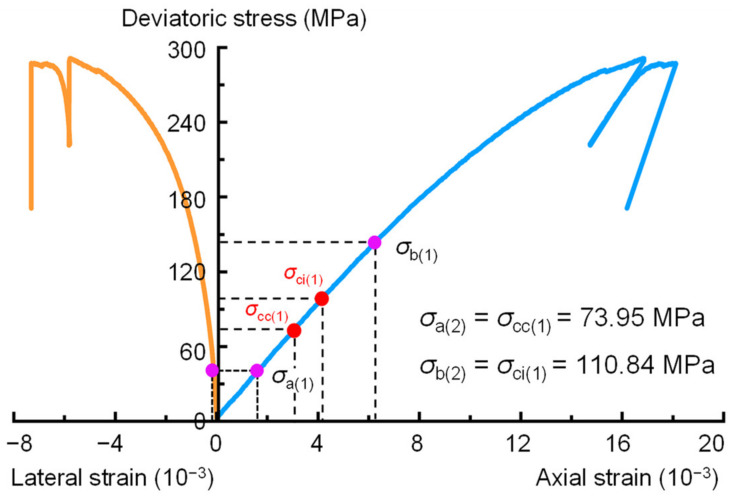
Definition of *σ*_a(2)_ and *σ*_b(2)_ for the calculation of the crack closure and initiation stresses of the 2000-0 sample using the BIA method.

**Figure 13:**
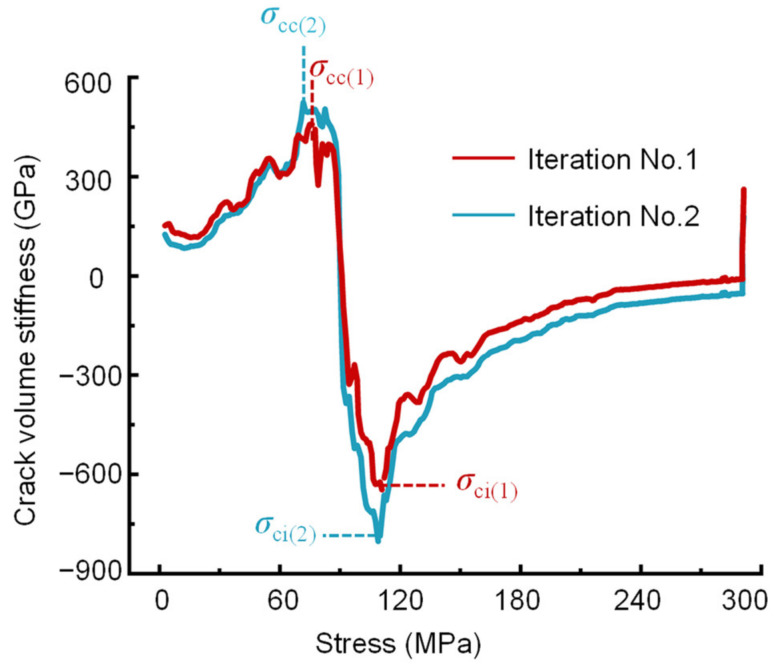
The procedure used to determine the crack closure stress and crack initiation stress for each iteration.

Poisson’s ratio and elastic modulus are two controlling elastic parameters that determine the brittleness of shale reservoirs, which defines the potential interval for hydraulic fracturing [3]. It is important to accurately determine the Poisson’s ratio and elastic modulus of shale. Therefore, the BIA method was proposed in Section 2 to determine the Poisson’s ratio and elastic modulus of the tested shale. Table 3 shows the calculated results of sample 2000-0. The tolerable errors of the crack closure and initiation thresholds are set to 1% for each iteration. For the initial trial, *σ*_a(1)_ is equal to the stress corresponding to 0.01% of the lateral strain, and *σ*_b(1)_ = 0.5*σ*_p_ (Figure 11). Table 3 shows that the crack closure stress *σ*_cc(1)_ = 72.95 MPa is notably larger than *σ*_a(1)_ (where *σ*_a(1)_ = 37.30 MPa), so *χ*_cc(1)_ > 0. The crack initiation stress *σ*_ci(1)_ = 110.84 MPa is significantly smaller than half of the peak stress (where *σ*_b(1)_ = 145.62 MPa), so *χ*_ci(1)_ < 0. That is, the shale sample of 2000-0 did not behave completely elastically within the stress range of *σ*_a(1)_ to *σ*_b(1)_. Consequently, the second iteration of the calculation needs to be performed. When *χ*_cc(1)_ > 0 and *χ*_ci(1)_ < 0, the range of *σ*_cc(1)_ to *σ*_ci(1)_ is closer to the real elastic stage, so *σ*_a(2)_ = *σ*_cc(1)_ = 73.95 MPa and *σ*_b(2)_ = *σ*_ci(1)_ = 110.84 MPa, as shown in Figure 12. This means that for the second iteration, the lower limit of the stress range for elastic parameter determination was taken as *σ*_cc(1)_, and the upper limit of the stress range was taken as *σ*_ci(1)_. After two iterations, the determined crack closure stresses *σ*_cc(1)_ and *σ*_cc(2)_ are 73.95 and 73.31 MPa, respectively, and *σ*_ci(1)_ and *σ*_ci(2)_ are 110.84 and 109.24 MPa, respectively. In addition, Figure 13 shows the procedure used to determine the crack closure stress and crack initiation stress for each iteration. The values of *χ*_cc(2)_ and *χ*_ci(2)_ calculated using Equations (5) and (6) are 0.49% and −0.96%, respectively. Both of these values are less than the tolerable error thresholds (*χ*_cc_ = *χ*_ci_ = 1%), satisfying the condition in Equations (7) and (8). The results indicate that the calculated elastic parameters are reliable after the 2nd iteration. Thus, the determined elastic modulus, Poisson’s ratio, crack closure stress, and crack initiation stress for the 2000-0 sample are *E* = 22.49 GPa, *ν* = 0.16, *σ*_cc_ = 73.31 MPa, and *σ*_ci_ = 109.78 MPa, respectively.

**Figure 14:**
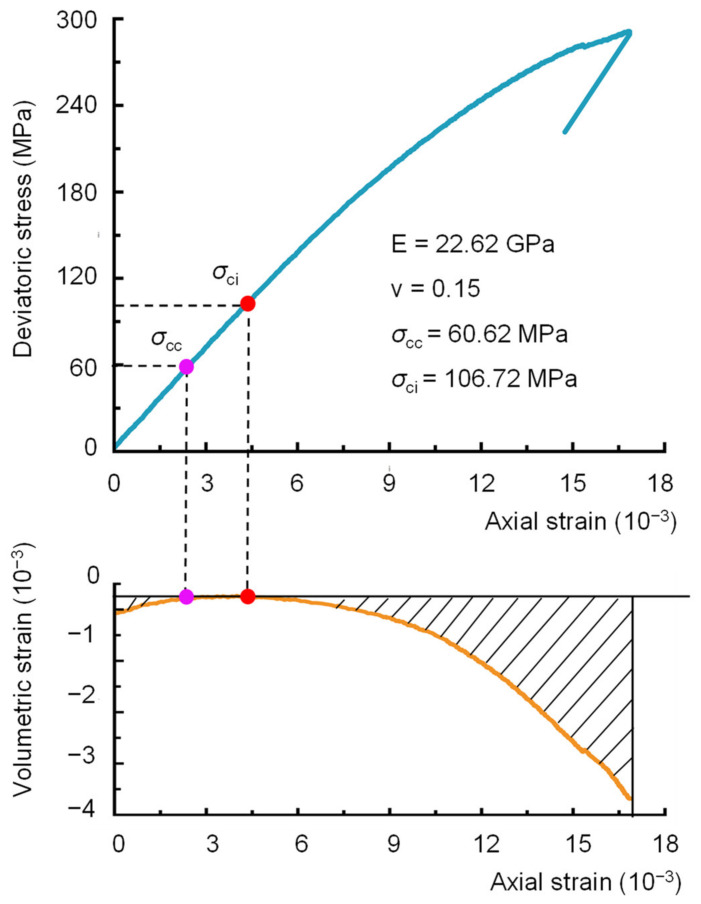
The elastic parameters, crack closure stress, and crack initiation stress were determined with the CVS method.

**Figure 15:**
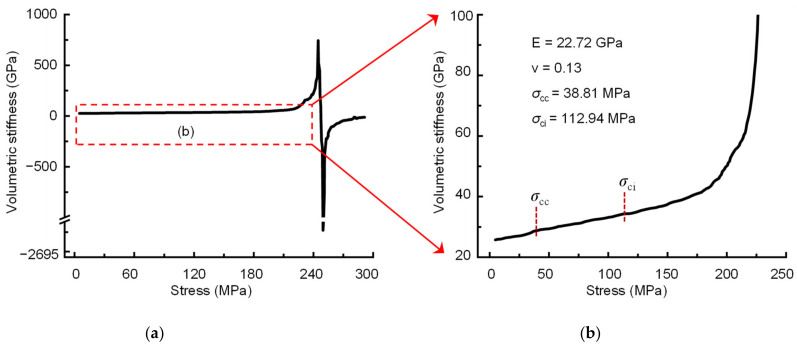
The elastic parameters, crack closure stress, and crack initiation stress determined by the VSM. (**a**) The stress-volumetric stiffness curve of sample 2000-0, and (**b**) is a localized magnification from 0–240 MPa in (**a**).

For comparison, the elastic parameters, crack closure stress and crack initiation stress of sample 2000-0 were also determined using three other methods: the crack volumetric strain method [28], the results for which are shown in Figure 14; the iterative method presented by He et al. [6], the results for which are shown in Table 4; and the VSM [29], the results for which are shown in Figure 15. The results of these three methods are shown in Table 5. It can be seen that the elastic moduli of sample 2000-0 calculated by the newly presented BIA method and the other three methods do not show marked differences. From the stress-strain curves shown in Figure 10, the relationship between the axial stress and the axial strain of sample 2000-0 is almost linear; therefore, the elastic moduli calculated by the four methods are similar. However, there is a significant difference in Poisson’s ratio between the four methods. Crack closure stresses obtained by the CVS method and VSM are significantly different from those obtained by the BIA method. Table 4 shows the iteration process for the iterative method, in which the crack closure stresses remained fixed at each iteration. There are obvious nonlinearities between the axial stress and the transverse strain of the 2000-0 sample, and the fixed crack closure stresses during the iterations cause inaccurate Poisson’s ratio measurements. This indicates that always using the stress corresponding to 0.01% of the lateral strain as the crack closure stress has a great effect on the accuracy of Poisson’s ratio, and further confirms the necessity for the BIA method to iteratively approach the linear region of the sample from both the upper and lower limit stresses. The above findings indicate that it is important to accurately obtain both the closure stress and the crack initiation stress in the process of determining the elastic parameters.

As mentioned above, accurate determination of both the closure stress and the crack initiation stress is important in accurately determining the elastic parameters. To investigate the effectiveness of the BIA method, experimental data obtained by Kim et al. [36] were adopted for further validation. Kim et al. [36] used an acoustic emission method to determine the *σ*_cc_ and *σ*_ci_ of typical granite under uniaxial compression, resulting in 56.20 MPa and 98.20 MPa, respectively. The *σ*_cc_ and *σ*_ci_ obtained by the BIA method are 57.87 MPa and 97.17 MPa, respectively. The results showed a close agreement between the crack closure stress and crack initiation stress values obtained by the BIA method and those obtained by the acoustic emission method. In addition, the BIA method can be applied to rectangular samples, and a comparison of results obtained from circular versus rectangular samples using the BIA method would be valuable. Further studies investigating the effectiveness of the BIA method on different sample shapes are recommended.

**Figure 16:**
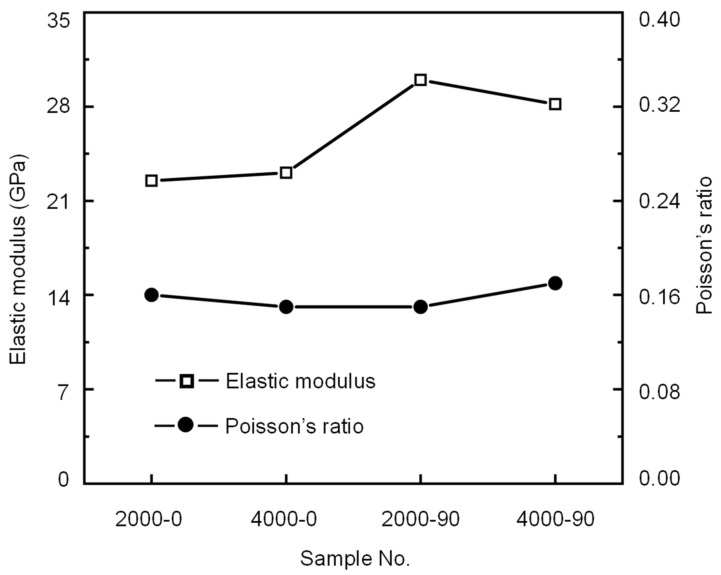
Variations in the elastic modulus and Poisson’s ratio of shale samples under different bedding plane orientations and depths.

Table 6 and Figure 16 show the elastic parameters of the shale samples with different bedding orientations and depth conditions calculated by the BIA method. The results show that for shale samples with the same bedding plane orientation, the change in the elastic modulus does not exceed 6% despite the increase in depth from 2000 m to 4000 m. A possible explanation is that the original cracks inside the shale are compacted under the confining pressure at a depth of 2000 m. Even as the confining pressure continues to increase at a depth of 4000 m, the elastic modulus of the compacted shale sample—as an index to measure the difficulty of a sample to generate elastic deformation—no longer increases. When the shale samples are at the same depth, the elastic modulus of the shale sample with *β* = 0° is approximately 20% less than that of the sample with *β* = 90°, which may be due to the strength of the bedding planes being significantly lower than that of the shale matrix. The results suggest that in deep conditions, the elastic modulus of the shale is more influenced by the bedding plane orientation than by the depth.

Poisson’s ratio is an elastic constant that reflects the lateral deformation of the shale. As shown in Table 6, the Poisson’s ratios of the 2000-0, 4000-0, 2000-90, and 4000-90 shale samples are 0.16, 0.15, 0.15, and 0.17, respectively. It can be seen in Figure 16 that the Poisson’s ratios of the studied shale samples are very similar, which is in agreement with the results obtained by [37,38] that there was little difference in Poisson’s ratio between shale samples at 0° and 90° under uniaxial compression tests. This result indicates that the Poisson’s ratios of shale samples with *β* = 0° and *β* = 90° are very similar, even under different geological conditions. From the uniaxial compression tests under room temperature conditions, the elastic modulus of the shale samples with *β* = 0° and *β* = 90° is in the range of 25~27 MPa and 35~38 MPa, respectively; Poisson’s ratio is in the range of 0.17~0.21 and 0.25~0.31, respectively [6,26,37,39]. The elastic modulus and Poisson’s ratio under the 2000 m and 4000 m depth conditions are smaller than those in the uniaxial compression tests under room temperature conditions. This indicates that the deep environment will change the mechanical properties of shale and that the effect of the depth on the mechanical properties of shale cannot be ignored.

### 3.3. Characteristic Stress Thresholds

**Figure 17:**
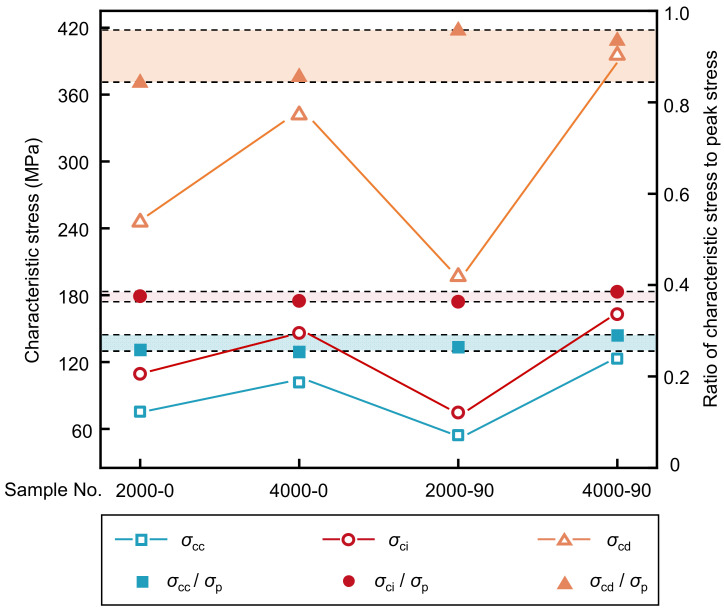
Variations in the characteristic stress of shale samples under different bedding plane orientations and depths.

The characteristic stresses (i.e., the crack closure stress *σ*_cc_, crack initiation stress *σ*_ci_, and crack damage stress *σ*_cd_) are determined from the stress-strain curves, which are important for better understanding the mechanical behavior of the rock [40,41,42]. The terms “crack initiation” and “crack damage” are used to describe the onset of damage (initiation) and the onset of crack coalescence to form macroscopic fractures (coalescence) [30,42,43]. In this study, the characteristic stresses of the shale samples were calculated using the BIA method, as shown in Table 7. Furthermore, to analyze the fracture process of shale samples with different depths and bedding plane orientations, the ratios of *σ*_cc_, *σ*_ci_, and *σ*_cd_ to the peak deviatoric stress (*σ*_cc_/*σ*_p_, *σ*_ci_/*σ*_p_, and *σ*_cd_/*σ*_p_) are shown in Figure 17.

#### 3.3.1. Crack Closure and Initiation Stresses

The results indicate that the crack closure stress (*σ*_cc_) and crack initiation stress (*σ*_ci_) are significantly affected by the bedding plane orientation of the samples and the simulated depth conditions of the test. Due to the restraining effect of the confining pressure on rock cracking, *σ*_cc_ and *σ*_ci_ increase continuously with increasing depth. When the depth is 2000 m, the *σ*_cc_ and *σ*_ci_ of the *β =* 0° sample are greater than those of the *β =* 90° sample. However, at a depth of 4000 m, the *σ*_cc_ and *σ*_ci_ of the *β =* 0° sample are smaller than those of the *β =* 90° sample. This result indicates that the effects of bedding plane orientation on *σ*_cc_ and *σ*_ci_ will change with changing depth. For the studied shale samples, the *σ*_cc_/*σ*_p_ ratios range from 24.83% to 25.16%, and the *σ*_ci_/*σ*_p_ ratios range from 34.78% to 38.23%, indicating that the *σ*_cc_/*σ*_p_ and *σ*_ci_/*σ*_p_ ratios do not change much and are less affected by the studied depth conditions and bedding plane orientations than the ratio of crack damage stress to peak deviatoric stress. Therefore, *σ*_ci_/*σ*_p_ can provide a reference for predicting the strength and failure stages of shale. Moreover, under a confining pressure of 60 MPa and room temperature conditions, the *σ*_ci_/*σ*_p_ values of the 0° and 90° shale samples are approximately 80% [25], which are significantly larger than those of the values obtained in the studied shale samples (34.78%~38.50%). This may be due to the uneven expansion of mineral particles in shale with increasing temperature, which makes the shale more susceptible to cracking under high-temperature in situ conditions.

#### 3.3.2. Crack Damage Stress

The determination of damage stress *σ*_cd_ is of great value for the long-term safety and stability analysis of large-scale projects [44,45,46]. Therefore, from the axial strain–volumetric strain curve, the volumetric strain reversal point can be used to determine *σ*_cd_, which is relatively accurate with low subjectivity. The *σ*_cd_ values of the shale samples obtained from the axial strain–volumetric strain curves are shown in Table 7. The characteristic stresses (*σ*_cc_, *σ*_ci_, and *σ*_cd_) increase with increasing depth from 2000 m to 4000 m for the shale samples with the same bedding plane orientations, as shown in Figure 17. Among them, the increase in *σ*_cd_ is significantly larger than that in *σ*_cc_ and *σ*_ci_, indicating that *σ*_cd_ is more sensitive to the change in depth, and that the increase in depth has an obvious inhibitory effect on crack extension. In addition, the *σ*_cd_/*σ*_p_ values of the *β* = 90° samples are higher than those of the *β =* 0° samples, which may be due to the high confining pressure inhibiting the crack extension of the *β =* 90° samples. Furthermore, the *σ*_cd_/*σ*_p_ values of the *β =* 90° samples are close to 1, indicating that the shale samples fail rather quickly after entering the unstable crack propagation stage without early warning. Therefore, in this case, the shale fracture and destruction process must be monitored in advance to ensure the safety of shale gas production.

### 3.4. Fracture Patterns

**Figure 18:**
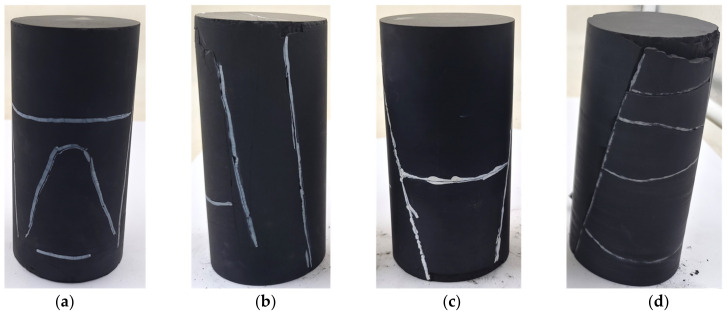
Failure morphology of samples: (**a**) 2000-0; (**b**) 2000-90; (**c**) 4000-0; (**d**) 4000-90.

From a microscopic viewpoint, rock failure is the result of the initiation, coalescence, and propagation of numerous microcracks [47]. Therefore, the fracture patterns reflect the stress state and physical properties of rock samples. Figure 18 shows the morphology of the fracture planes of the studied shale samples. For all the shale samples studied, the morphology of failure planes showed a thoroughgoing shear fracture along diagonal and transverse cracks parallel or perpendicular to the bedding planes. However, under uniaxial compression tests, the failure mechanisms of the shale samples with *β* = 0° failed by shear sliding, while the samples with *β* = 90° exhibited a splitting failure along weak bedding [37,48]. This result shows that the in situ geological conditions have a great influence on the failure mechanisms of shale samples, so the effect of deep in situ conditions on shale mechanism properties cannot be ignored. In addition, the degree of damage and damage characteristics are not identical for different samples due to the variability in the in situ and bedding plane orientation. When the depth is 2000 m, a subshear surface crack can be observed on the *β* = 0° and *β* = 90° sample surfaces, and when the depth increases to 4000 m, the shale sample surfaces do not show any spalling phenomenon. This may be because as the depth increases, the damage to the sample surface by spalling is inhibited. Meanwhile, for the *β* = 90° shale samples, shear cracks across the diagonal of the samples split the samples completely, and transverse cracks through the shale matrix break the samples completely. However, neither shear cracks nor cracks along the bedding planes penetrated the *β* = 0° shale samples. As a result, the *β* = 90° shale samples are more fragmented than the *β* = 0° shale samples at depths of 2000 m and 4000 m, respectively. In addition, the 4000-90 samples are penetrated by shear cracks, with half of the samples being split into differently sized clasts by transverse cracks perpendicular to the loading direction through the shale matrix.

## 4. Conclusions

Several triaxial compression experiments at high temperatures and confining pressures were performed to simulate in situ conditions at 2000 m and 4000 m depths on Longmaxi shale samples with two typical bedding plane orientations (perpendicular and parallel to the axial loading direction). In addition, a new BIA method for determining crack closure and crack initiation thresholds was proposed to accurately determine the elastic modulus and Poisson’s ratio. The elastic parameters, characteristic stresses, and fracture patterns of Longmaxi shale under triaxial compression at different simulated depths were analyzed. The main conclusions are as follows:

(1) The peak deviatoric stresses for the 2000-0, 4000-0, 2000-90, and 4000-90 samples are 291.35, 399.47, 205.51, and 422.97 MPa, respectively. As depth increases from 2000 to 4000 m, the peak deviatoric stresses of shale samples also increase. Furthermore, the increase was greater for samples with *β* = 90° than for those with *β* = 0°. The results indicate that the strength of shale samples with *β* = 90° is more sensitive to changes in deep in situ conditions than samples with *β* = 0°.
(2) For all the shale samples studied, thoroughgoing shear fractures along bedding planes were observed in the fracture morphology. At a depth of 2000 m, subshear surface and spalling failure were observed, whereas no spalling phenomenon was observed at a depth of 4000 m. The reason may be that the spalling of the sample surface is inhibited as the depth increases.
(3) The Poisson’s ratios of the 2000-0, 4000-0, 2000-90, and 4000-90 shale samples are 0.16, 0.15, 0.15, and 0.17, respectively, and their elastic moduli are 22.49, 23.08, 29.99, and 28.19 MPa, respectively. It can be seen that Poisson’s ratios of the studied shale samples are very similar. However, the elastic moduli and Poisson’s ratios of studied shale samples are both smaller than those obtained under room temperature and pressure conditions. This indicates that the in situ deep conditions will change the mechanical properties of shale, and the effect of the depth on the mechanical properties of shale cannot be ignored.
(4) For the studied shale samples, the *σ*_cc_/*σ*_p_ ratios range from 24.83% to 25.16%. The *σ*_ci_/*σ*_p_ ratios range from 34.78% to 38.23%, indicating that the *σ*_cc_/*σ*_p_ and *σ*_ci_/*σ*_p_ ratios do not change much and are less affected by both the studied depth conditions and bedding plane orientations than the ratio of crack damage stress to peak deviatoric stress. In addition, as the depth increases from 2000 to 4000 m, the increase in *σ*_cd_ is significantly larger than that of *σ*_cc_ and *σ*_ci_, indicating that *σ*_cd_ is more sensitive to changes in depth, and the increase in depth has an obvious inhibitory effect on crack extension.

## Figures and Tables

**Table 1:** Crack closure and initiation thresholds of the 4000-0 shale sample with different Poisson’s ratios.

Passion’s Ratio	*σ*_cc_ (MPa)	*σ*_ci_ (MPa)
*ν* = 0.15	98.83	142.20
*ν* = 0.20	182.98	232.69
*ν* = 0.25	248.95	275.37

**Table 2:** The temperatures and confining pressures applied correspond to the simulated in situ conditions at different depths.

Sample No.	Depth (m)	Bedeeing Plane Orientation (°)	Temperature (°C)	Confining Pressure (MPa)
2000-0	2000	0	73.49	42.64
2000-90		90	73.49	42.64
4000-0	4000	0	102.70	89.15
4000-90		90	102.70	89.15

**Table 3:** The elastic parameters, crack closure stress, and crack initiation stress of sample 2000-0 were determined with the BIA method.

Iteration No.	Elastic Modulus (GPa)	Poisson’s Ratio	Crack Closure Stress *σ*_cc_ (MPa)	Crack Initiation Stress *σ*_ci_ (MPa)
1	22.34	0.16	72.95	110.84
2	22.49	0.16	73.31	109.78

**Table 4:** The elastic parameters, crack closure stress, and crack initiation stress of sample 2000-0 were determined with the iterative method.

Iteration No.	Elastic Modulus (GPa)	Poisson’s Ratio	*σ*_cc_ (MPa)	*σ*_ci_ (MPa)
0 (initial)	22.34	0.16	37.30	145.62
1	22.81	0.13	37.30	106.72
2	22.99	0.12	37.30	94.53
3	22.80	0.12	37.30	94.34

**Table 5:** The elastic parameters, crack closure stress, and crack initiation stress of sample 2000-0 were determined with different methods.

Method	Elastic Modulus (GPa)	Poisson’s Ratio	*σ*_cc_ (MPa)	*σ*_ci_ (MPa)
BIA method	22.49	0.16	73.31	109.78
CVS method	22.62	0.15	60.62	106.72
Iterative method	22.80	0.12	37.30	94.34
VSM	22.72	0.13	38.81	112.94

**Table 6:** Elastic parameters of the studied shale determined using various methods.

Specimen No.	Elastic Modulus (GPa)	Poisson’s Ratio
2000-0	22.49	0.16
4000-0	23.08	0.15
2000-90	29.99	0.15
4000-90	28.19	0.17

**Table 7:** Characteristic stresses of shale samples with different bedding plane orientations and different depth conditions.

Specimen No.	*σ*_p_ (MPa)	*σ*_cc_ (MPa)	*σ*_cc_/*σ*_p_ (%)	*σ*_ci_ (MPa)	*σ*_ci_/*σ*_p_ (%)	*σ*_cd_ (MPa)	*σ*_cd_/*σ*_p_ (%)
2000-0	291.35	73.31	25.16	109.78	37.68	245.35	84.21
4000-0	399.47	99.94	25.02	138.94	34.78	341.62	85.52
2000-90	205.51	51.03	24.83	74.79	36.39	196.61	95.67
4000-90	422.97	122.63	28.99	161.7	38.23	395.11	93.41

## Data Availability

No new data were created or analyzed in this study. Data sharing is not applicable to this article.
